# Stunting Is Associated with Food Diversity while Wasting with Food Insecurity among Underfive Children in East and West Gojjam Zones of Amhara Region, Ethiopia

**DOI:** 10.1371/journal.pone.0133542

**Published:** 2015-08-18

**Authors:** Achenef Motbainor, Alemayehu Worku, Abera Kumie

**Affiliations:** School of Public Health College of Health Sciences, Addis Ababa University, Addis Ababa, Ethiopia; TNO, NETHERLANDS

## Abstract

**Background:**

Food insecurity has detrimental effects in protecting child undernutrition.This study sought to determine the level of child undernutrition and its association with food insecurity.

**Methods:**

A community based comparative cross-sectional study design involving multistage sampling technique was implemented from 24^th^ of May to 20^th^ of July 2013. Using two population proportion formula, a total of 4110 randomly selected households were included in the study. Availability of the productive safety net programme was used for grouping the study areas. A multiple linear regression model was used to assess the association between food insecurity and child malnutrition. Clustering effects of localities were controlled during analysis.

**Results:**

Stunting (37.5%), underweight (22.0%) and wasting (17.1%) were observed in East Gojjam zone, while 38.3% stunting, 22.5% underweight, and 18.6% wasting for the West Gojjam zone. Food insecurity was significantly associated with wasting (β = - 0.108, P < 0.05).Food diversity and number of meals the child ate per day significantly associated with stunting (β = 0.039, P < 0.01) and underweight (β = 0.035, P < 0.05) respectively. Residential area was the significant predictor of all indices.

**Conclusion:**

The magnitude of child undernutrition was found to be very high in the study areas. Food insecurity was the significant determinant of wasting. Food diversity and number of meals the child ate per day were the significant determinants of stunting and underweight respectively. Child nutrition intervention strategies should take into account food security, dietary diversity, and carefully specified with regard to residential locations. Addressing food insecurity is of paramount importance.

## Introduction

Food insecurity is a state or a condition in which people experienced limited or uncertain physical and economic access to safe, sufficient, and nutritious food to meet their dietary needs or food preferences for a productive, healthy, and active life [1–3]. Food insecurity and associated malnutrition result in serious health problems and loss of human potential for economic developments in developing countries [3]. Food insecurity causes not only potentially serious health consequences like hanger and acute malnutrition but also complications such as hypertension, hyperlipidemia and reduced health and quality of life [1, 4].

Food insecurity remains highly prevalent in developing countries, and over the past two decades, it has increasingly been recognised as a serious public health problem in the developed world [4].

Food and Agriculture Organization (FAO) recently estimated that globally about 842 million people were incapable of getting their dietary energy requirements in 2012/13 report. This showed that around one in eight people in the world are likely to have suffered from chronic hunger, not having enough food for an active and healthy life. The vast majority of hungry people -827 million of them- live in developing regions, where the prevalence of undernourishment is now estimated at 14.3% in 2011–13 [5].

Different studies in different parts of the world showed that level of food insecurity is still very high and need special attention. A study in low-income households of Los Angeles, for instance, showed that the food insecurity level was 24.4% [4]. In Canada, a study done to differentiate the socio-demographic risk factors between Aboriginal and non-Aboriginal households revealed that 33% of Aboriginal households were food insecure compared with 9% of non-Aboriginal households [6].

The scale of food insecurity in developing countries is still significantly higher than that of developed. In Kenya, a study conducted in 2011 showed, of all households involved, 70.1% categorized as severely food insecure, 21.9% moderately food insecure, and 3.7% were mildly food insecure, whereas 4.3% were food secure [3].

According to astudy done in Fartaworeda, Ethiopia, in 2012, from the total study participants about 70.7% were categorized as food insecure [7]. Similarly, a study done in Addis Ababa city showed that 58.16% of the total households were below the food insecurity line [8]. Another longitudinal study in Ethiopia on adolescents’ food security status showed that different levels of food insecurity resulted in different rounds of the study period. Overall, 20.5% of adolescents were food insecure in the first round survey, while the proportion of adolescents with food insecurity increased to 48.4% one year later. During the one year follow up period, more than half (54.8%) of the youth encountered transient food insecurity [9].

In Ethiopia, the magnitude ofboth food insecurity and child undernutrition is very high. Different study results in different parts of the country showed various but high levels of malnutrition prevalences. The 2011 EDHS result showed that the prevalence of stunting was 44% [10]. According to a study done in West Gojjam zone stunting, wasting and underweight accounted for 43.2%, 14.8%, 49.2% respectively [11]. Another study done in Dollo Ado district, Somali region showed the level of child undernutritionin the country was 42.3% wasting, 34.4% stunting, and 47.7% underweight showed the level of child undernutrition in the country[11, 12].

Although there are variations from region to region and locality to locality, a relevant literature review showed that inappropriate feeding practice, maternal undernutrition, household food insecurity, economic growth and maternal education are varied and intertwined principal determinants of child nutrition [13]. Study results in Bangladesh showed that household food insecurity was a significant determinants of child undernutrition in the form of stunting (OR = 1.62), underweight (OR = 1.80) and wasting (OR = 1.28) [14]. Another study result from eight different countries of Asia and Africa showed that household food insecurity was the significant determinants of child nutrition [15].

Other studies did not find association between household food insecurity and child undernutrition. For example, study done in Kenya found no association between household food insecurity and child undernutrition while it found significant association between stunting and severe food insecurity [16]. Similarly, a study in Ghana showed that household food insecurity was not associated with child undernutrition while it found that food diversity significantly determined stunting [17].

Productive safety net program is an intensive and innovative starategy the Ethiopian government run so far to respond chronic food insecurity problems in a systematic manner [18]. The program targeted poor and food insecure households [19].

With the presence of this high level of child undernutrition, intensive works to reduce these problems by designing different programmes such as National Nutrition Strategy [20] and the disparities, it is appropriate to evaluate and determine the level of child undernutrition and moreover, the effect of food insecurity on child malnutrition. Therefore, the purpose of this study was to determine the level of child malnutrition by comparing the two populations that have been classified as areas with and without productive safety net programme and in addition it assessed the association of food insecurity with child nutrition status.

## Materials and Methods

### Study area and period

The study was conducted in Amhara Regional State that covers some 157,647 km^2^ across north western and eastern Ethiopia and having a total projected population of 20,018,999 (10,011,795 males and 10,007,204 females) based on 2007 census [21, 22]. The region is divided in to a number of highland blocks separated by deep river valleys and the eastern and western escarpments and their associated lowlands [22]. Specifically the study was conducted in Eastand West Gojjam zones of the region. East Gojjam zone has 2,451,959 total populations (1,199,952 males and 1,252,006 females), while West Gojjam zone has a total population of 2,474,254 (1,220,477 males and 1,253,777 females) [21]. The mean annual temperature of the region ranges from 22–27°C in the lowlands, and between 10 and 22°C in places that have height of up to 3,000 meter above sea level [22]. Four major cereal systems have been recognized in the region: sorghum-maize system in the lowland agro-ecological zone, wheat-teff system in the single rain season area of the mid-land agro-ecological zone, wheat-teff system in the double rain seasons of the mid-land agro-ecological zone and barley system in the high land agro-ecological zone [22]. This study considered two zones classified based on their agricultural production: areas with production safety net programme and without the programme.

### Study design, sample size determination and sampling techniques

Community based comparative cross-sectional study design was used to determine the levels of food insecurity, malnutritionand the association between the two. Households in the study area were used as a sampling unit and all the necessary data were drawn from the under five children and their mothers. Two groups were considered based on their food production and availability of safety net programme: group 1 was without safety net programme and group 2 was with safety net programme. The prevalence of stunting was taken into account to determine the sample size which is considered as the best indicator of nutritional status of the community and also since it is not affected by acute events. The 2011 EDHS national prevalence (44%) [10]indicate for food surplus area and 50% (because no specific study and since it gives the possible maximum sample size) for food insecure area, were used as the malnutrition prevalence. Using two population proportion formula and the difference at the significance level of 1% and power of 90%, 4110 total households were randomly selected and included in the study.

Multistage sampling technique was implemented to reach and select the final study units. In the first place, the two zones (East and West Gojjam) were selected purposely by taking into account the food security variability in the two zones. Woredas (districts) in East Gojjam zone are considered as food insecure area while the West Gojjam zone is considered as food surplus area by the regional government. Six woredas from the two zones (three from each zone) covered by safety net program from East Gojjam zone (EnebsieSarMidir, GonchaSisoEnesie, and ShebelBerenta) were selected and included in the study. From a total of 14 woredas in West Gojam zone, three woredas (Mecha, North Achefer, and JabiTehinan) were choosen. The two zones are more comparable in many socio-cultural characteristics than the other zones of the region.

Once the woredas were identified, kebeles were randomly selected from the selected woredas. The kebeles (the smallest administrative unit in the country) were selected based on agroecological zones and urban rural settings. Four town kebeles, three rural highland kebeles, eleven rural midland kebeles, and six rural lowland kebeles were selected randomly.

Then, the total sample size was distributed to the kebele proportionally. The households from these kebeles were selected using systematic random sampling technique. The total number of households in each kebele was divided by the allocated sample size to get the sampling interval. When there were no under five children in the identified household, the next household was used as sampling unit. When there was more than one mother with under five children in the same household, one mother was selected by lottery method.

### Data collection tools and techniques

Socio-demographic data were collected by structured questionnaire adapted from different standard questionnaires. The anthropometric data were collected using the procedure stipulated by the WHO (1995) for taking anthropometric measurements. Household food security access information was collected by using the questionnaire developed by Food and Nutrition Technical Assistant Project (FANTA) [23]. The questionnaire was pre-tested in a similar setting after translating into the local language, Amharic. The equipments that were used to measure the anthropometric variables were calibrated each day prior to the actual data collection by using a known weight material. The data collectors and supervisors were university graduate BSc holders.

#### Height

For height, a vertical or horizontal measuring board reading a maximum of 175cm and capable of measuring to 0.1cm was used to take the height of a child. The child stands on the measuring board barefooted; have hands hanging loosely with feet parallel to the body, and heels, buttocks, shoulders and back of the head touching the board. The head would be held comfortably erect with the lower border of the orbit of the eye being in the same horizontal plane as the external canal of the ear. The headpiece of the measuring board is then pushed gently, crushing the hair and making contact with the top of the head. Height is then read to the nearest 0.1cm. Two readings were recorded and the computed average was used in the analysis.

#### Length

For children aged to 24 months, length instead of height was taken. The child was made to lie flat on the length board. The sliding piece is placed at the edge of the bare feet as the head (with crushing of the hair) touches the other end of the measuring device. Then two readings were taken and the average was computed.

#### Weight

An easily portable weighing scale, graduated by 0.1 kg, was used. The scale was adjusted before weighing every child by setting it to zero. The child was lightly dressed during having the weight taken. Two readings were taken for each child and the average was recorded on the questionnaire.

#### Mid Upper Arm Circumference (MUAC)

MUAC was measured on the left arm, at the midpoint between the elbow and the shoulder. The arm was relaxed and hanging down the side of the body. A MUAC measuring tape was placed around the arm. The value was read from the window of the tape without pinching the arm or leaving the tape loose.

### Dependent variables

Stunting

Underweight

Wasting

Middle upper arm circumference (MUAC)

### Operational definitions

**Food/dietary diversity**- defined as the number of different foods or food groups consumed over a given reference period [16].

**Food insecurity**- is a state or a condition in which people experienced limited or uncertain physical and economic access to safe, sufficient and nutritious food to meet their dietary needs or food preferences [3].

**Food security**- A situation when all people at all times have both physical and economic access to safe, sufficient and nutritious food to meet their dietary needs or food preferences for a productive, healthy and active life [3, 23].

**Stunting, chronic malnutrition**–reflects long term cumulative effects of inadequate nutrition and health. Shortness in height refers to low height-for-age that may reflect either normal variation in growth or a deficit in growth. It is defined as low height-for-age at< -2 SD of the median value of the NCHS/WHO international growth reference [24].

**Underweight**–an anthropometric index of weight-for-age represents body mass relative to age. Defined as low weight-for-age at < -2 SD of median value of the NCHS/WHO international growth reference [24].

**Wasting, acute malnutrition**- a nutritionally deficient state of recent onset related to sudden food deprivation or malabsorption or poor utilization of nutrients which result in rapid weight losts. Wasting refers to low weight-for-height at < -2 SD of median value of the NCHS/WHO international growth reference [24].

### Data quality control

To assure the quality of the data and to makeall assessment team members able to administer the questionnaires properly, a total of five days rigorous training of enumerators and supervisors was provided. Before the actual data collection began, data collectors and supervisors carried out roleplay practices and then field pre-test activities. At the end of every data collection day, each questionnaire was examined for its completeness and consistency by the field supervisors and the principal investigator and pertinent feedback was given to the data collectors and supervisors.

### Data management and analysis

The data were coded, entered and cleaned by Epi-Info version 3.5.3 and exported to SPSS version 20 for further analysis. Descriptive summaries such as frequencies, proportions, percentages, mean, standard deviationsand prevalence were determined. For underfive children WHO Anthroversion 3.2.2 software was used to enter and determine the prevalence of malnutrition. To identify predictor variables for child nutritional status, multiple linear regression model was employed using the entire method, by considering the Z-score values as a continous outcome of interest. To check the assumptions of linearity and model fitness, scatter plot of standardized residuals against standardized predicted value, and the normality probability plot (P-P plot) was used. Multicollinearity diagnosis was implemented by using variance inflation factor (VIF) and Durbin-Watson value for independence. The clustering effect of localities (kebeles) was controlled during analysis.

### Ethical clearance

Ethical clearance was obtained from the Institutional Review Board (IRB) of the College of Health Sciences of Addis Ababa University and Amhara Regional Health Bureau. Participation in the study was on a voluntary basis and written consent, signed or verified by fingerprint, was obtained from study participants. Parents/care givers of the children were informed about the study and written consent on behalf of the children were obtained from the parents. The consent form was attached to each questionnaire and parents signed before the measurement and the interview. The consent form was approved by the IRB during the ethical clearance process. Privacy and confidentiality were maintained. Sick and severely malnourished children were advised and referred to the nearest health facilities and nutritional advice was given to parents.

## Results

### Socio-demographic characteristics

From the total 4110 visited households, 3964 respondents (with a response rate of 96.45%) gave complete responses. One thousand nine hundred eighty five (50.1%) of respondents were from East Gojjam and the rest 1979 (49.9%) were from West Gojjam. The majority (85.5%) of the household heads were men. Similarly 3416(86%) of the respondents were rural residents. Sixty nine point six percent of the fathers were farmers and 1.6% lived on different activities such as tailor, weaver, blacksmith, carpenter, church servant, wood work and pension. Similarly, about 1.9% of mothers were local beer and liquor sellers and students (Table 1). The agro ecological zone distribution of the respondents was 363 (9.2%) from the highlands (*Dega*) elevation of > 2400m above sea level, 2373 (59.9%) from medium highland (*WoynaDega*) elevation of 1500 – 2400m, and 1228 (31%) from the lowland (*Kola*) elevation of < 1500m above sea level.

**Table 1:** Socio-demographic characteristics of the study participants (n = 3964) in East and West Gojjam zones of Amhara region, 2013.

Variables	Number (n)	Percent (%)
**Household head:**		
Female	568	14.3
Male	3396	85.7
**Marital status:**		
Married	3410	86
Divorced	388	9.8
Widowed	103	2.6
Separate	27	0.7
Single	36	0.9
**Family size:**		
2–4	1872	47.2
≥ 5	2092	52.8
**Maternal educational level:**		
No formal education	3281	82.8
Have a formal education	683	17.2
**Paternal educational level:**		
No formal education	2527	72
Have a formal education	984	28
**Maternal occupation:**		
Housewife	1807	45.6
Farmer	1591	40.1
Private organization employs	21	0.5
Merchant	433	10.9
Government employ	75	1.9
Daily labourer	99	2.5
Others	74	1.9
**Paternal occupation:**		
Farmer	2758	69.6
Merchant	483	12.2
Private organization employee	64	1.6
Daily labourer	118	3
Government employee	143	3.6
Others	63	1.6
**Residential place:**		
Rural	3416	86.2
Urban	548	13.8

### Child nutritional status

The mean (SD) age of children was 28.02 (14.45) months. There were 1746 (44%) female and 2218 (56%) male children participated in the study. One thousand forty four (26.34%) children were in 24–35 months age groups and 3.53% children were in 0–5 months age groups.

The total prevalence of child malnutrition were37.6%, (95% CI: 36.5, 39.3) stunting, underweight 21.9% (95% CI: 20.9, 23.5) and 17.3% (95% CI: 16.8, 19.0)for wasting (Table 2).

**Table 2:** Anthropometric distribution of children by age and sex, east and west Gojjam zones of Amhara region, 2013.

Age group in month	N	WAZ				HAZ				WHZ			
		% < -3 SD	% <- 2 SD*	Mean	SD	% <- 3 SD	% <- 2 SD*	Mean	SD	% <- 3 SD	% <- 2 SD*	Mean	SD
**Combined sexes**													
**Total (0–60)**	**3964**	**5.6**	**21.9**	**-1.1**	**1.15**	**14.2**	**37.6**	**-1.42**	**1.42**	**6.2**	**17.3**	**-0.46**	**1.58**
(0–5)	140	7.9	22.1	-0.89	1.35	15	36.4	-1.12	1.59	7.9	17.9	-0.05	1.98
(6–11)	463	8.9	30.2	-1.29	1.22	14.7	38.7	-1.48	1.42	7.6	23.3	-0.58	1.74
(12–23)	958	7.3	27.8	-1.26	1.2	18.7	47.8	-1.62	1.5	6.6	20.1	-0.65	1.58
(24–35)	1044	5.7	23.2	-1.14	1.15	15.7	40.2	-1.53	1.36	9	18	-0.48	1.66
(36–47)	803	3.6	15.2	-0.91	1.07	10.7	29.6	-1.23	1.37	3.7	13.4	-0.3	1.44
(48–60)	556	2.2	12.4	-0.9	0.98	8.1	26.1	-1.14	1.34	2.3	11.7	-0.32	1.31
**Females**													
**Total (0–60)**	**1746**	**5.5**	**18.7**	**-0.93**	**1.17**	**13.6**	**35.3**	**-1.35**	**1.42**	**4.6**	**14.4**	**-0.25**	**1.6**
(0–5)	73	6.8	16.4	-0.59	1.33	17.8	34.2	-1.22	1.57	2.7	12.3	0.5	1.89
(6–11)	214	7.9	20.1	-0.98	1.2	10.3	31.3	-1.22	1.4	7	19.2	-0.36	1.79
(12–23)	377	4	21.2	-1.01	1.17	13.5	40.3	-1.4	1.48	4.8	15.6	-0.44	1.6
(24–35)	477	7.3	19.9	-0.94	1.18	18.9	40.3	-1.56	1.37	5.2	13.4	-0.14	1.63
(36–47)	363	4.4	16.3	-0.88	1.17	11.3	31.7	-1.25	1.4	3.6	12.7	-0.22	1.45
(48–60)	242	3.3	15.7	-0.94	1.05	8.3	26.9	-1.18	1.34	3.3	13.6	-0.34	1.4
**Males**													
**Total (0–60)**	**2218**	**5.7**	**24.5**	**-1.22**	**1.12**	**14.7**	**39.4**	**-1.46**	**1.42**	**7.4**	**19.6**	**-0.62**	**1.55**
(0–5)	67	9	28.4	-1.22	1.31	11.9	38.8	-1.02	1.61	13.4	23.9	-0.65	1.93
(6–11)	249	9.6	39	-1.55	1.18	18.5	45	-1.7	1.4	8	26.9	-0.77	1.68
(12–23)	581	9.5	32	-1.42	1.19	22	52.7	-1.76	1.5	7.7	23.1	-0.78	1.56
(24–35)	567	4.2	25.9	-1.3	1.1	13.1	40.2	-1.5	1.35	12.2	21.9	-0.76	1.64
(36–47)	440	3	14.3	-0.93	0.97	10.2	28	-1.21	1.35	3.9	14.1	-0.37	1.43
(48–60)	314	1.3	9.9	-0.86	0.93	8	25.5	-1.12	1.34	1.6	10.2	-0.3	1.23

* < -2 SD includes < -3 SD

### Association of food insecurity with child nutritional status

Socio-demographic variables such as residential area and child sex significantly associated with wasting (β = 0.149 and 0.109, P < 0.05 respectively). When a child becomes urban resident, the WHZ-scoreincreasedon average by 0.149 units compared to rural residents. Similarly, the WHZ-score of female children increased on average by 0.109 units compared to male children. Age, sex and residential area were also the significant predictors of child MUAC (β = 0.017, 0.078 and 0.441, P < 0.001 respectively).

Food insecurity associated significantly with wasting (β = - 0.108 at P < 0.05) but it was not found to be the significant predictor of stunting and underweight. Food diversity and residential area were significant predictors of stunting (β = 0.039, P < 0.05) (Table 3). A 1 unit increased in food diversity predicted on average 0.039 (95% CI: 0.007, 0.070) increased in child height-for-age. Residential areawas significantly associated with WAZ- score (β = 0.185, P< 0.001). If a child is from urban resident, the WAZ-score increased on average by 0.185 units compared to a child from rural resident. Number of meals per day the child ate was also found to be significant determinants of WAZ-score (β = 0.035, P < 0.05). When the number of meals the child ate per day increased by 1 unit, the weight of the child will increase by 0.035kg. Food diversity and number of meals the child ate per day predicted child MUAC (β = 0.027 and 0.141, P < 0.001 respectively) (Table 4). This showed that when the number of meals the child ate per day increased by 1 unit, the MUAC of the child increased by 0.141cm.

**Table 3:** Linear regression analysis of selected variables relating to child nutritional status (HAZ, WHZ and WAZ) in east and west Gojjam zones of Amhara Region, 2013.

Variables	Height-for-age (stunting)		
	β	Se of β	95% CI
Constant	-1.250	0.185	(- 1.612, -0.888)
Age	0.002	0.002	(- 0.001, 0.005)
Sex	- 0.022	0.046	(- 0.111, 0.068)
Residential area	0.162	0.068	(0.029, 0.294)**
Agroecology	0.010	0.039	(- 0.065, 0.086)
Family size	0.010	0.046	(- 0.080, 0.099)
Food insecurity	- 0.039	0.048	(- 0.029, 0.065)
Number of meals per day	0.013	0.022	(- 0.029, 0.056)
Food diversity	0.039	0.016	(0.007,0.070)**
	**Weight-for-height (wasting)**		
Constant	- 0.676	0.205	(- 1.078,- 0.274)
Age	0.003	0.002	(- 0.001, 0.006)
Sex	0.109	0.051	(0.010, 0.208)*
Residential area	0.149	0.075	(0.002, 0.296)*
Agroecology	- 0.030	0.043	(-0.114, 0.054)
Family size	-0.119	0.051	(-0.219,- 0.020)
Food insecurity	- 0.108	0.053	(- 0.213,-0.004)*
Number of meals per day	0.037	0.024	(- 0.010, 0.085)
Food diversity	0.017	0.018	(- 0.019, 0.052)
	**Weight-for-age (under weight)**		
Constant	-1.148	0.149	(-1.441,- 0.856)
Age	0.003	0.001	(0.000, 0.005)
Sex	0.066	0.037	(-0.006, 0.138)
Residential area	0.185	0.055	(0.078, 0.292)***
Agroecology	-0.012	0.031	(-0.073, 0.049)
Family size	-0.079	0.037	(-0.152,- 0.007)*
Food insecurity	0.054	0.039	(-0.022, 0.130)
Number of meals per day	0.035	0.018	(0.001, 0.070)*
Food diversity	- 0.012	0.013	(- 0.038, 0.013)

* P < 0.05

** P < 0.01

*** P < 0.001

**Table 4:** Linear regression analysis of selected variables relating to child nutritional status (MUAC) in east and west Gojjam zones of Amhara Region, 2013.

Variables	Child Middle Upper Arm Circumference (MUAC)		
	β	Se of β	95% CI
Constant	13.170	0.145	(12.886, 13.445)
Age	0.017	0.001	(0.014, 0.019)***
Sex	0.078	0.036	(0.008, 0.148)*
Residential area	0.441	0.053	(0.337, 0.542)***
Agroecology	- 0.175	0.030	(- 0.235,- 0.116)***
Family size	- 0.063	0.036	(- 0.134, 0.007)
Food insecurity	- 0.057	0.038	(- 0.017, 0.131)
Food diversity	0.027	0.013	(0.002, 0.052)*
Number of meals per day	0.141	0.017	(0.107, 0.174)***

* P < 0.05

** P < 0.01

*** P < 0.001

## Discussion

The nutritional status of underfive children is affected by different interrelated broad range of factors. This study aimed to determine the level of undernutrition and the effect of household food insecurity on the nutritional status of underfive children. Finding from this study result showed that there was high level of child malnutrition in the study areas. Althoughthere was a decrease in stunting and underweight prevalence as compared to a previous EDHS result (44.1% and 29% respectively), the prevalenceis still significant [10]. The study also showed that malnutrition prevalence was decreased as compared to study results in different places of the country; West Gojjam zone (stunting and underweight, 43.2% and 49.2% respectively), Dollo Ado district, Somali region, Ethiopia (underweight, 47.7%)[11, 12], South East Amhara, Ethiopia (stunting and underweight 60.6% and 31.1% respectively) [25], Sidama, South Ethiopia (underweight 25.6%) [26]. The study result is less prevalence than base line study results of Ethiopia conducted by alive and thrive project (stunting 50.7%, underweight 27.5%)[27] and the general undernutrition prevalence of Eastern Africa sub region (stunting 45.3%) [28]. On the other hand, the magnitude of acute malnutrition (wasting) was higher than the recent EDHS (9%), West Gojjam zone study results (14.8%) [10, 11], Sidama, South Ethiopia (8.3%)[26], and base line study results of Ethiopia conducted by alive and thrive project [27].

According to the WHO malnutrition classification, when stunting, underweight, and wasting become more than 40%, 30% and 15% it is considered as very high or critical and high or serious when stunting, underweight and wasting become in the range of 30–39.9%, 20–29.9% and 10–14.9% in the community respectively [29]. Therefore, the prevalence of malnutrition (stunting and underweight) in the study area was in the serious range and wasting was in the critical range. Compared to findings from the previous studies, there is a reduction in both stunting and underweight. However, the rate wasfound to be in the serious range. Even so, the figures are differ markedly from the figure expected to achieve the MDG and World Food Summit (WFS) by 2015 (below 15%) [5].

The study also determined the association of food insecurity and other determinants with child malnutrition. The finding showed that there was no substantial difference in child nutritional status between the two study areas. Food insecurity was found to be a significant determinants of wasting but not associated with stunting and underweight. Results from different studies reported both the presence and absence of significant association between food insecurity and malnutrition. Studies done in rural Bangladesh and Colombia showed the significant association between food insecurity and malnutrition [14, 30, 31]. Other studies that are consistent with the current study that tested the presence of an association between the two variables after adjusting other confounding variables showed the absence of association [17, 32, 33].

While there are substantial evidences indicating that household food security is among the key determinants of nutritional status of children [34], and food security may be a necessary prerequisite for good nutrition outcomes, it is insufficient by its own [35]. The influence of food security in the nutritional status of children can be affected by other determinants such as mother knowledge on child nutrition and health care practices, maternal nutritional status, intrahousehold food allocation and utilization practices and access to health services and healthy environmental conditions. For instance, intra-family food distribution is often related to hierarchical position with the head of the family receiving priority in eating while mothers and children receiving a smaller share of the family’s food relative to their nutritional need [36]. It is quite similar in the study area community to prepare and feed children the essential child nutrition. Culturally the community does not give attention to child nutrition and simply feed one common kind of food (*injera and wat*) because of lack of awareness regarding balanced diets. It is also a common trend in the study community to eat meat and meat products only during holiday and party occasions. It could be explained by these facts that most malnutrition indices were common in West Gojjam zones than East Gojjam zone while food insecurity was higher in East Gojjam zone.

The other circumstances that should be considered in child nutrition include; prelacteal feeding, introduction of other diet before six months, initiation of complementary diet after one years of age (too late or too early complementary diet initiation) [37].This indicates that it is not lack or shortage of food that predisposes young children to malnutrition, but also lack of knowledge of appropriate infant and young child feeding practices. Studies done in Kersa Demographic Surveillance and Health Research Centre, Ethiopia and Vietnam, showed that exclusive breast feeding was a significant determinants of child undernutrition [38, 39]. Ananother study done in Democratic Republic of Congo revealed the presence of high malnutrition in households that produced food products probably because of the economic decision to sell more than the population consume [40]. It is also common practice in the study area to sell important cereals and legumes (peas, chickpeas and beans), including milk and milk products since they earn better income by selling these agricultural products rather than feeding these nutritious food items to their children.

In line with the above explanations, food diversity or dietary diversity and number of meals the child ate per day significantly associated with stunting, underweight and MUAC of the child [17, 41]. The number of meals the child ate per day and the food diversity (the number of food items that grouped in to cereals, fruits, milk and milk products, poultry, legumes, vegetables, meat, roots and tubers, oil and fats, sugar and honey, fish and sea animals and species) are taken in to account as an important practice for child feeding when the mother knows about the importance of child nutrition rather than the households become food secure[42].According to study result done in Nepal, children fed complementary food less than four times a day were 3.60 times more likely to be malnourished than their counter parts (95% CI: 1.32–9.95)[41].

Residential area was the other significant determinants of child undernutrition in the current study. It is supported by other study done in Vietnam that showed the presence of significant association between residential area and undernutrition [39]. This could be due to the fact that mothers in the urban setting have more access to nutrition related information that could be disseminated through different mass media and such information enables urban mothers to feed their child properly compared to their counterparts.

## Conclusions and Recommendations

The research findings from this study showed that, there was a high level of child malnutrition and different forms of multidisciplinary and interrelated determinants for malnutrition. Therefore, all forms of malnutrition intervention program conducted in the country should focus on different aspects of child care and feeding practices. Firstly, the food insecurity intervention programme should be strengthened to bring about fundamental improvements on both economic development and child malnutrition. Child malnutrition intervention strategies should be focused not only on food security programs but also on diversity of agricultural products that are basic elements of food diversity and food items to provide the child with balanced and nutritious food. Besides food insecurity intervention and food diversity actions for child nutrition, the number of foods the child ate per day require special attention. For proper child feeding practices and provide optimum numbers of meals per day and associated diversity virtual, mother knowledge on nutrition is important. Hence, to reduce child malnutrition, awareness campaigns aimed at educating the importance of child nutrition and more appropriate feeding practices should be designed and implemented. The nutritional intervention measures should also be carefully specified with regard to residential localities. Moreover, further research is needed to investigate infant and young child feeding practices and agricultural product preservation mechanisms.
